# Supplementary figures and images for: Designed and validated novel allele-specific primer to differentiate Kernel Row Number (KRN) in tropical field corn
Source: PLoS One. 2023 Apr 12;18(4):e0284277. doi: 10.1371/journal.pone.0284277 (PMC10096290; doi:10.1371/journal.pone.0284277)

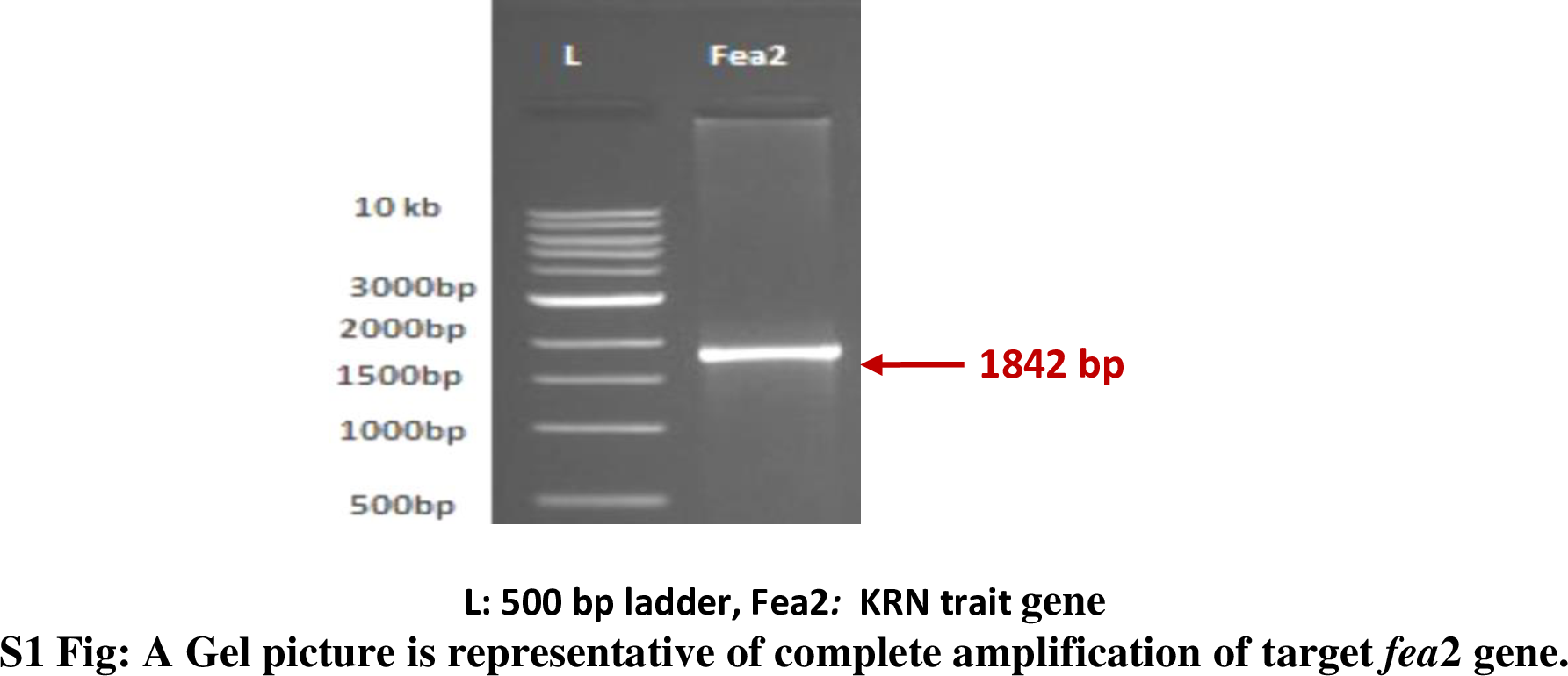

Supplement: S1 Fig — (TIF) [file pone.0284277.s001.tif]

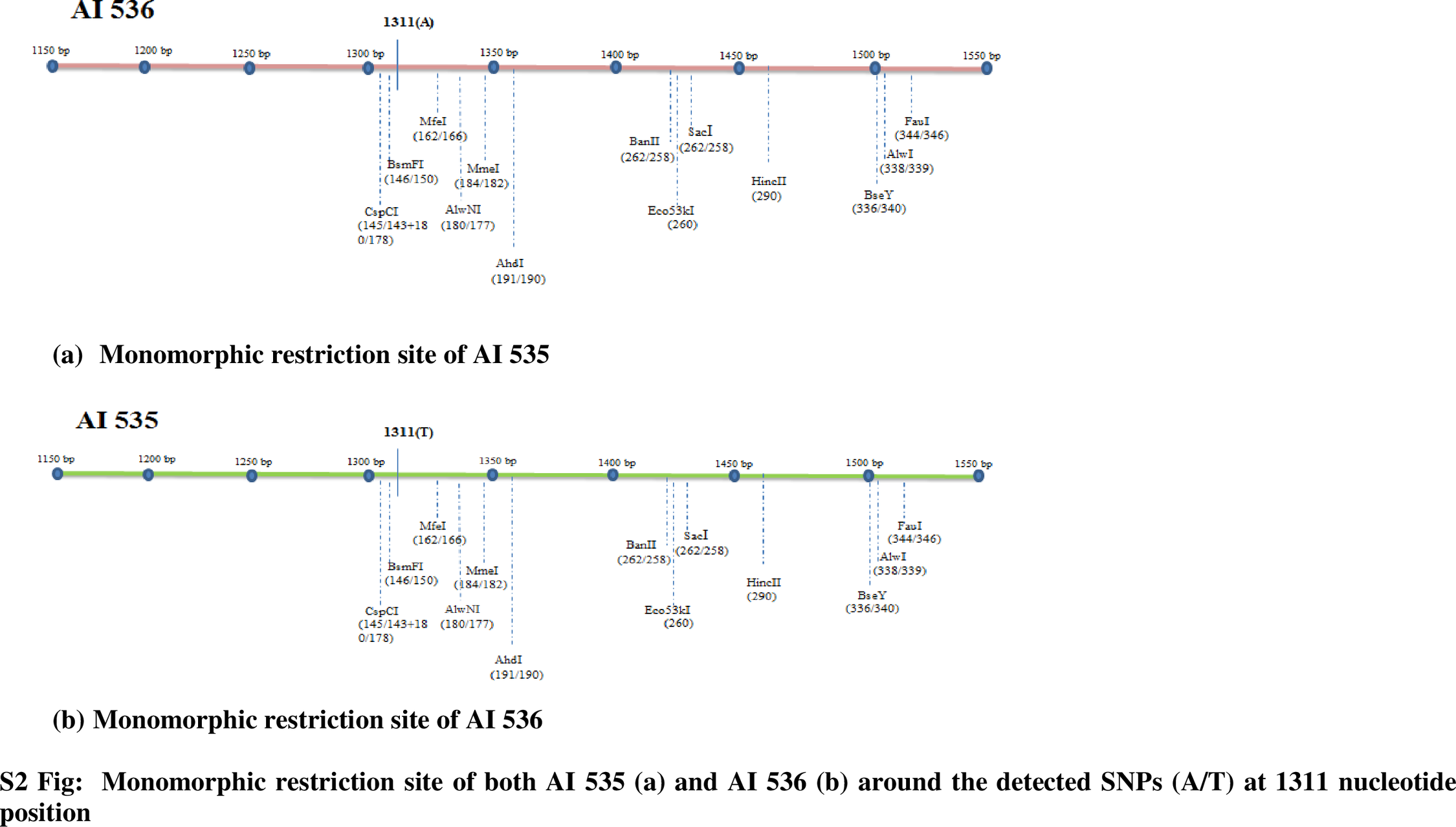

Supplement: S2 Fig — Monomorphic restriction site of both AI 535 (a) and AI 536 (b) around the detected SNPs (A/T) at 1311 nucleotide position. (a) Monomorphic restriction site of AI 535. (b) monomorphic restriction site of AI 536. (TIF) [file pone.0284277.s002.tif]

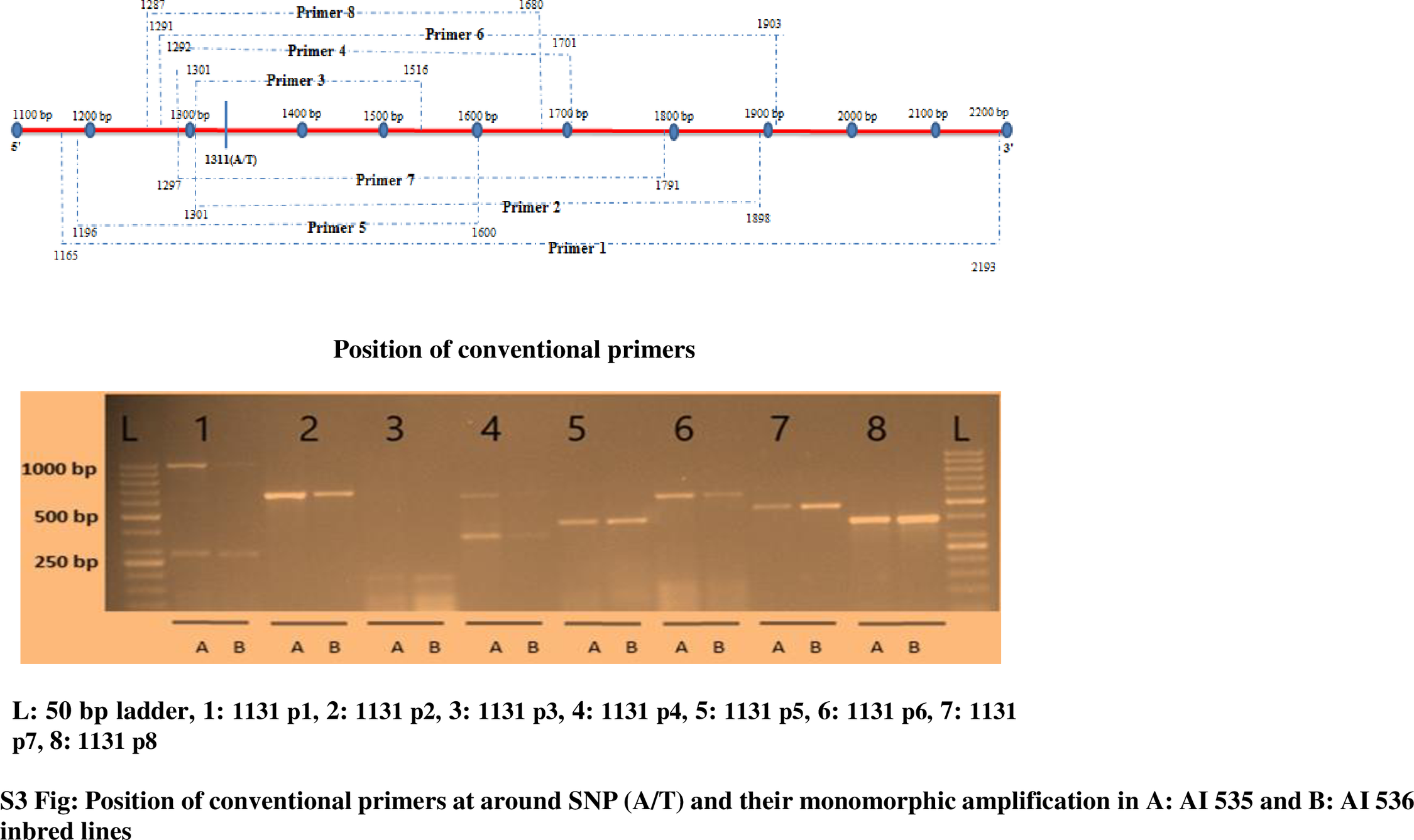

Supplement: S3 Fig — Position of conventional primers at around SNP (A/T) and their monomorphic amplification in A: AI 535 and B: AI 536 inbred lines. (TIF) [file pone.0284277.s003.tif]
