# Supplementary material for: Designed and validated novel allele-specific primer to differentiate Kernel Row Number (KRN) in tropical field corn
Source: PLoS One. 2023 Apr 12;18(4):e0284277. doi: 10.1371/journal.pone.0284277 (PMC10096290; doi:10.1371/journal.pone.0284277)
Supplement: S1 Table — (DOCX) [file pone.0284277.s004.docx]

**S1 Table: Mean yield, IPCA1, IPCA2, ASVi and ASVi rank for genotypes over three locations:**

| **Genotype** | **Mean** | **IPCA1** | **IPCA2** | **ASVi** | **ASVi rank** | **Genotype** | **Mean** | **IPCA1** | **IPCA2** | **ASVi** | **ASVi rank** |
| --- | --- | --- | --- | --- | --- | --- | --- | --- | --- | --- | --- |
| **AI01** | 3.017 | -0.223 | 0.073 | 4.83 | 21 | **AI24** | 3.035 | 0.235 | -0.087 | 5.08 | 24 |
| **AI02** | 3.225 | -0.173 | 0.087 | 3.74 | 14 | **AI25** | 3.297 | 0.020 | 0.437 | 0.61 | 4 |
| **AI03** | 3.015 | -0.470 | -0.031 | 10.17 | 40 | **AI26** | 2.577 | 0.265 | 0.079 | 5.73 | 29 |
| **AI04** | 2.872 | -0.004 | -0.023 | 0.09 | 1 | **AI27** | 2.877 | 0.129 | -0.096 | 2.80 | 10 |
| **AI05** | 2.482 | -0.086 | -0.100 | 1.85 | 7 | **AI28** | 3.07 | -0.429 | 0.179 | 9.28 | 39 |
| **AI06** | 1.367 | -0.382 | -0.180 | 8.26 | 36 | **AI29** | 2.095 | 0.289 | 0.027 | 6.26 | 31 |
| **AI07** | 3.163 | -0.166 | 0.091 | 3.59 | 13 | **AI30** | 2.59 | -0.190 | 0.016 | 4.10 | 17 |
| **AI08** | 2.427 | 0.117 | 0.039 | 2.52 | 9 | **AI31** | 1.97 | 0.602 | 0.156 | 13.02 | 44 |
| **AI09** | 3.543 | -0.207 | -0.150 | 4.48 | 19 | **AI32** | 3.365 | -0.252 | 0.148 | 5.44 | 26 |
| **AI10** | 3.547 | -0.229 | -0.240 | 4.97 | 22 | **AI33** | 2.942 | 0.213 | 0.102 | 4.61 | 20 |
| **AI11** | 2.618 | -0.231 | 0.024 | 5.01 | 23 | **AI34** | 2.878 | 0.300 | 0.041 | 6.49 | 34 |
| **AI12** | 2.64 | 0.134 | 0.090 | 2.90 | 11 | **AI35** | 2.722 | 0.033 | 0.019 | 0.72 | 5 |
| **AI13** | 3.095 | 0.061 | -0.209 | 1.33 | 6 | **AI36** | 3.07 | -0.498 | -0.032 | 10.76 | 41 |
| **AI14** | 2.983 | 0.150 | 0.074 | 3.25 | 12 | **AI37** | 3.593 | 0.014 | 0.032 | 0.31 | 2 |
| **AI15** | 3.112 | -0.545 | 0.098 | 11.79 | 42 | **AI38** | 2.25 | 0.633 | 0.034 | 13.70 | 45 |
| **AI16** | 2.587 | 0.417 | 0.034 | 9.03 | 38 | **AI39** | 3.495 | -0.249 | -0.013 | 5.38 | 25 |
| **AI17** | 3.568 | -0.255 | -0.092 | 5.52 | 27 | **AI40** | 3.55 | -0.110 | 0.024 | 2.37 | 8 |
| **AI18** | 2.615 | -0.017 | -0.087 | 0.37 | 3 | **AI41** | 3.597 | -0.382 | -0.047 | 8.27 | 37 |
| **AI19** | 1.933 | 0.256 | 0.015 | 5.55 | 28 | **AI42** | 3.345 | -0.571 | -0.003 | 12.34 | 43 |
| **AI20** | 1.928 | 0.294 | -0.092 | 6.37 | 32 | **AI43** | 3.125 | 0.185 | 0.051 | 4.01 | 16 |
| **AI21** | 2.655 | 0.200 | -0.133 | 4.34 | 18 | **AI44** | 3.05 | 0.268 | -0.147 | 5.79 | 30 |
| **AI22** | 2.693 | 0.378 | -0.229 | 8.17 | 35 | **AI45** | 2.835 | 0.298 | -0.314 | 6.46 | 33 |
| **AI23** | 2.702 | 0.175 | 0.335 | 3.79 | 15 |  |  |  |  |  |  |

IPCA: Interaction Principal Component Analysis, ASV: Average Stability Value
