# Supplementary material for: Designed and validated novel allele-specific primer to differentiate Kernel Row Number (KRN) in tropical field corn
Source: PLoS One. 2023 Apr 12;18(4):e0284277. doi: 10.1371/journal.pone.0284277 (PMC10096290; doi:10.1371/journal.pone.0284277)
Supplement: S2 Table — (DOCX) [file pone.0284277.s005.docx]

**S2 Table: Monomorphic restriction site present both in AI 535 and AI 536 at 440 bp (1161) around the 1311nucleotide position (inclusive) in the *fea2* gene**

| **S.No.** | **Restriction Enzymes** | **Restriction sites** | **Position** |
| --- | --- | --- | --- |
| 1 | AhdI | GACNNNNNGTC | 191/190 |
| 2 | AlwNI | CAGNNNCTG | 180/177 |
| 3 | AlwI | GGATCNNNNN | 338/339 |
| 4 | BanII | GRGCYC | 262/258 |
| 5 | BseYI | CCCAGC | 336/340 |
| 6 | BsmFI | GGGAC(N)10NNNN | 146/150 |
| 7 | CspCI | NN(N)11CAA(N)5GTGG(N)10NN | 145/143+180/178 |
| 8 | Eco53kI | GAGCTC | 260 |
| 9 | FauI | CCCGCNNNNNN | 344/346 |
| 10 | HincII | GTYRAC | 290 |
| 11 | MfeI | CAATTG | 162/166 |
| 12 | MmeI | TCCRAC(N)18NN | 184/182 |
| 13 | SacI | GAGCTC | 262/258 |
