# Supplementary material for: Designed and validated novel allele-specific primer to differentiate Kernel Row Number (KRN) in tropical field corn
Source: PLoS One. 2023 Apr 12;18(4):e0284277. doi: 10.1371/journal.pone.0284277 (PMC10096290; doi:10.1371/journal.pone.0284277)
Supplement: S3 Table — (DOCX) [file pone.0284277.s006.docx]

**S3 Table: Pedigree of inbred lines used in the experimentation**

| S.No. | Inbred lines | Pedigree | S.No. | Inbred lines | Pedigree | S.No. | Inbred lines | Pedigree |
| --- | --- | --- | --- | --- | --- | --- | --- | --- |
| 1 | AI 501 | MDR 9-1-2-1-B-B-B | 16 | AI 516 | MDR 119-6-4-2-2-2-B | 31 | AI 531 | MDR 358-1-4-2-2-B-B |
| 2 | AI 502 | MDR 9-4-2-1-1-B-B | 17 | AI 517 | MDR 145-2-2-2-2-B | 32 | AI 532 | MDR 359-3-2-2-B-B |
| 3 | AI 503 | MDR 12-1-1-1-B-B-B | 18 | AI 518 | MDR 154-1-1-B-B | 33 | AI 533 | (VQL-1 X VQL-2) 341-1-1-B-B |
| 4 | AI 504 | MDR 41-3-2-2-B-B-B | 19 | AI 519 | MDR 177-1-5-2-B-B | 34 | AI 534 | JH-13249-138-1-1-B-B |
| 5 | AI 505 | MDR 54-3-2-2-B-B | 20 | AI 520 | MDR 183-1-1-1-B-B | 35 | AI 535 | PML 31-1-2-1-B-B-B |
| 6 | AI 506 | MDR 62-3-1-1-B-B-B | 21 | AI 521 | MDR 194-1-1-1-B-B | 36 | AI 536 | PML 60-1-1-1-B-B |
| 7 | AI 507 | MDR 65-3-2-2-B | 22 | AI 522 | MDR 208-2-1-1-B-B | 37 | AI 537 | (BML6XCE18)-74-19-2-1-1-B |
| 8 | AI 508 | MDR 67-4-1-1-B | 23 | AI 523 | MDR 250-1-1-1-B-B | 38 | AI 538 | DML 2070-2-1-1-B |
| 9 | AI 509 | MDR 80-1-2-2-B | 24 | AI 524 | MDR 263-3-2-2-2-B | 39 | AI 539 | P-3501-10-1-3-1-B-B |
| 10 | AI 510 | MDR 82-1-1-1-B-B | 25 | AI 525 | MDR 291-3-1-1-B | 40 | AI 540 | MDR 264-4-5-5-2-B-B |
| 11 | AI 511 | MDR 82-3-1-1-1-B-B | 26 | AI 526 | MDR 292-4-2-2-B-B | 41 | AI 541 | SAFAL-X12-9-1-1-B-B |
| 12 | AI 512 | MDR 95-2-1-1-B-B | 27 | AI 527 | MDR 299-1-2-2-B-B | 42 | AI 542 | KDMH-755-12-1-2-B-B |
| 13 | AI 513 | MDR 111-1-2-2-B-B-B | 28 | AI 528 | MDR 318-2-1-1-B-B | 43 | AI 543 | RMH-3591-1-1-1-B-B |
| 14 | AI 514 | MDR 114-3-2-2-B-B | 29 | AI 529 | MDR 343-2-1-1-B-B | 44 | AI 544 | KDMH-176-5-1-1-B-B |
| 15 | AI 515 | MDR 119-2-2-1-B-B | 30 | AI 530 | MDR 348-1-24-2-2-B | 45 | AI 545 | P-3501-10-1-3-B-B |
