# Supplementary material for: Designed and validated novel allele-specific primer to differentiate Kernel Row Number (KRN) in tropical field corn
Source: PLoS One. 2023 Apr 12;18(4):e0284277. doi: 10.1371/journal.pone.0284277 (PMC10096290; doi:10.1371/journal.pone.0284277)
Supplement: S4 Table — (DOCX) [file pone.0284277.s007.docx]

**S4 Table : List of nonspecific primer(s) designed to target 1311nucleotide position of *fea*2 gene**

| **S.No** | **Primer** | **Forward primer** | **Reverse primer** | **Amplicon size** |
| --- | --- | --- | --- | --- |
| 1 | 1131 p1 | GCCACCTACCTGACTGGATGTTC | GTCCAATCCTCCAAACACGCCAG | 1029bp |
| 2 | 1131 p2 | TCCATCAGAGAGTGTGCTTCCACCCCAA | CACGAAGTTTCTTGCATTAGAGGAGC | 598 bp |
| 3 | 1131 p3 | CCATCAGAGAGTGTGCTTCCACC | GCAGGGATCTGCCCAGCCAAGTAAT | 215 bp |
| 4 | 1131 p4 | TCAGGGGACTCCATCAGAGAGTGTGC | CATTCTCAGAGCACCCTTTCCCACTGCA | 410 bp |
| 5 | 1131 p5 | CCTGCTCCAGTGGCTTGATTTGTCTAG | GCTGCAATTCCAGGAGGCACCTC | 405 bp |
| 6 | 1131 p6 | GTCAGGGGACTCCATCAGAGAGT | CGAAACACGAAGTTTCTTGCATTAG | 609 bp |
| 7 | 1131 p7 | GGACTCCATCAGAGAGTGTGCTTC | CATGCCAGCCACCAAGCCAAC | 495bp |
| 8 | 1131 p8 | GTGGTCAGGGGACTCCATCAGAGA | ATGCTCCTGGGAACTTCGTTGTTGGC | 393 bp |
