# Supplementary material for: Designed and validated novel allele-specific primer to differentiate Kernel Row Number (KRN) in tropical field corn
Source: PLoS One. 2023 Apr 12;18(4):e0284277. doi: 10.1371/journal.pone.0284277 (PMC10096290; doi:10.1371/journal.pone.0284277)
Supplement: S5 Table — (DOCX) [file pone.0284277.s008.docx]

**S 5 Table : Amplification status of 1311*fea*2.1 in F_2_ population (AH-4500 F_2_)**

| S.No. | Genotype | Kernel Row Number | Amplification status of 1311fea2.1 primer |
| --- | --- | --- | --- |
| 1 | AH-4500 F2-1 | 12 | - |
| 2 | AH-4500 F2-2 | 14 | - |
| 3 | AH-4500 F2-3 | 12 | - |
| 4 | AH-4500 F2-4 | 12 | - |
| 5 | AH-4500 F2-5 | 10 | - |
| 6 | AH-4500 F2-6 | 10 | - |
| 7 | AH-4500 F2-7 | 10 | - |
| 8 | AH-4500 F2-8 | 20 | + |
| 9 | AH-4500 F2-9 | 12 | - |
| 10 | AH-4500 F2-10 | 14 | - |
| 11 | AH-4500 F2-11 | 10 | - |
| 12 | AH-4500 F2-12 | 10 | - |
| 13 | AH-4500 F2-13 | 10 | - |
| 14 | AH-4500 F2-14 | 22 | + |
| 15 | AH-4500 F2-15 | 14 | - |
| 16 | AH-4500 F2-16 | 12 | - |
| 17 | AH-4500 F2-17 | 10 | - |
| 18 | AH-4500 F2-18 | 10 | - |
| 19 | AH-4500 F2-19 | 12 | - |
| 20 | AH-4500 F2-20 | 20 | + |
| 21 | AH-4500 F2-21 | 10 | - |
| 22 | AH-4500 F2-22 | 12 | - |
| 23 | AH-4500 F2-23 | 14 | - |
| 24 | AH-4500 F2-24 | 12 | - |
| 25 | AH-4500 F2-25 | 12 | - |
| 26 | AH-4500 F2-26 | 10 | - |
| 27 | AH-4500 F2-27 | 10 | - |
| 28 | AH-4500 F2-28 | 12 | - |
| 29 | AH-4500 F2-29 | 18 | + |
| 30 | AH-4500 F2-30 | 18 | + |
| 31 | AH-4500 F2-31 | 12 | - |
| 32 | AH-4500 F2-32 | 14 | - |
| 33 | AH-4500 F2-33 | 12 | - |
| 34 | AH-4500 F2-34 | 12 | - |
| 35 | AH-4500 F2-35 | 10 | - |
| 36 | AH-4500 F2-36 | 10 | - |
| 37 | AH-4500 F2-37 | 12 | - |
| 38 | AH-4500 F2-38 | 18 | + |
| 39 | AH-4500 F2-39 | 10 | - |
| 40 | AH-4500 F2-40 | 12 | - |
| 41 | AH-4500 F2-41 | 20 | + |
| 42 | AH-4500 F2-42 | 12 | - |
| 43 | AH-4500 F2-43 | 14 | - |
| 44 | AH-4500 F2-44 | 12 | - |
| 45 | AH-4500 F2-45 | 12 | - |
| 46 | AH-4500 F2-46 | 18 | + |
| 47 | AH-4500 F2-47 | 10 | - |
| 48 | AH-4500 F2-48 | 12 | - |
| 49 | AH-4500 F2-49 | 12 | - |
| 50 | AH-4500 F2-50 | 20 | + |
| 51 | AH-4500 F2-51 | 10 | - |
| 52 | AH-4500 F2-52 | 10 | - |
| 53 | AH-4500 F2-53 | 10 | - |
| 54 | AH-4500 F2-54 | 18 | + |
| 55 | AH-4500 F2-55 | 12 | - |
| 56 | AH-4500 F2-56 | 12 | - |
| 57 | AH-4500 F2-57 | 12 | - |
| 58 | AH-4500 F2-58 | 16 | + |
| 59 | AH-4500 F2-59 | 10 | - |
| 60 | AH-4500 F2-60 | 10 | - |
| 61 | AH-4500 F2-61 | 12 | - |
| 62 | AH-4500 F2-62 | 12 | - |
| 63 | AH-4500 F2-63 | 14 | - |
| 64 | AH-4500 F2-64 | 14 | - |
| 65 | AH-4500 F2-65 | 18 | + |
| 66 | AH-4500 F2-66 | 12 | - |
| 67 | AH-4500 F2-67 | 14 | - |
| 68 | AH-4500 F2-68 | 14 | - |
| 69 | AH-4500 F2-69 | 18 | + |
| 70 | AH-4500 F2-70 | 12 | - |
| 71 | AH-4500 F2-71 | 14 | - |
| 72 | AH-4500 F2-72 | 14 | - |
| 73 | AH-4500 F2-73 | 20 | + |
| 74 | AH-4500 F2-74 | 12 | - |
| 75 | AH-4500 F2-75 | 14 | - |
| 76 | AH-4500 F2-76 | 10 | - |
| 77 | AH-4500 F2-77 | 20 | + |
| 78 | AH-4500 F2-78 | 10 | - |
| 79 | AH-4500 F2-79 | 12 | - |
| 80 | AH-4500 F2-80 | 18 | + |
| 81 | AH-4500 F2-81 | 18 | + |
| 82 | AH-4500 F2-82 | 10 | - |
| 83 | AH-4500 F2-83 | 10 | - |
| 84 | AH-4500 F2-84 | 16 | + |
| 85 | AH-4500 F2-85 | 18 | + |
| 86 | AH-4500 F2-86 | 12 | - |
| 87 | AH-4500 F2-87 | 10 | - |
| 88 | AH-4500 F2-88 | 14 | - |
| 89 | AH-4500 F2-89 | 18 | + |
| 90 | AH-4500 F2-90 | 10 | - |
| 91 | AH-4500 F2-91 | 18 | + |
| 92 | AH-4500 F2-92 | 12 | - |
| 93 | AH-4500 F2-93 | 18 | + |
| 94 | AH-4500 F2-94 | 10 | - |
| 95 | AH-4500 F2-95 | 18 | + |
| 96 | AH-4500 F2-96 | 12 | - |
| 97 | AH-4500 F2-97 | 12 | - |
| 98 | AH-4500 F2-98 | 10 | - |
| 99 | AH-4500 F2-99 | 16 | + |
| 100 | AH-4500 F2-100 | 10 | - |
| 101 | AH-4500 F2-101 | 18 | + |
| 102 | AH-4500 F2-102 | 12 | - |
| 103 | AH-4500 F2-103 | 16 | + |
| 104 | AH-4500 F2-104 | 18 | + |
| 105 | AH-4500 F2-105 | 10 | - |
| 106 | AH-4500 F2-106 | 12 | - |
| 107 | AH-4500 F2-107 | 14 | - |
| 108 | AH-4500 F2-108 | 14 | - |
| 109 | AH-4500 F2-109 | 12 | - |
| 110 | AH-4500 F2-110 | 18 | + |
| 111 | AH-4500 F2-111 | 18 | + |
| 112 | AH-4500 F2-112 | 14 | - |
| 113 | AH-4500 F2-113 | 12 | - |
| 114 | AH-4500 F2-114 | 12 | - |
| 115 | AH-4500 F2-115 | 12 | - |
| 116 | AH-4500 F2-116 | 16 | + |
| 117 | AH-4500 F2-117 | 10 | - |
| 118 | AH-4500 F2-118 | 18 | + |
| 119 | AH-4500 F2-119 | 12 | - |
| 120 | AH-4500 F2-120 | 16 | + |
| 121 | AH-4500 F2-121 | 14 | - |
| 122 | AH-4500 F2-122 | 18 | + |
| 123 | AH-4500 F2-123 | 12 | - |
| 124 | AH-4500 F2-124 | 18 | + |
| 125 | AH-4500 F2-125 | 12 | - |
| 126 | AH-4500 F2-126 | 12 | - |
| 127 | AH-4500 F2-127 | 12 | - |
| 128 | AH-4500 F2-128 | 16 | + |
| 129 | AH-4500 F2-129 | 10 | - |
| 130 | AH-4500 F2-130 | 18 | + |
| 131 | AH-4500 F2-131 | 10 | - |
| 132 | AH-4500 F2-132 | 18 | + |
| 133 | AH-4500 F2-133 | 10 | - |
| 134 | AH-4500 F2-134 | 16 | + |
| 135 | AH-4500 F2-135 | 10 | - |
| 136 | AH-4500 F2-136 | 18 | + |
| 137 | AH-4500 F2-137 | 10 | - |
| 138 | AH-4500 F2-138 | 16 | + |
| 139 | AH-4500 F2-139 | 12 | - |
| 140 | AH-4500 F2-140 | 14 | - |
| 141 | AH-4500 F2-141 | 12 | - |
| 142 | AH-4500 F2-142 | 10 | - |
| 143 | AH-4500 F2-143 | 10 | - |
| 144 | AH-4500 F2-144 | 12 | - |
| 145 | AH-4500 F2-145 | 10 | - |
| 146 | AH-4500 F2-146 | 12 | - |
| 147 | AH-4500 F2-147 | 18 | + |
| 148 | AH-4500 F2-148 | 14 | - |
| 149 | AH-4500 F2-149 | 16 | + |
| 150 | AH-4500 F2-150 | 12 | - |
| 151 | AH-4500 F2-151 | 18 | + |
| 152 | AH-4500 F2-152 | 12 | - |
| 153 | AH-4500 F2-153 | 10 | - |
| 154 | AH-4500 F2-154 | 12 | - |
| 155 | AH-4500 F2-155 | 10 | - |
