# Supplementary material for: Designed and validated novel allele-specific primer to differentiate Kernel Row Number (KRN) in tropical field corn
Source: PLoS One. 2023 Apr 12;18(4):e0284277. doi: 10.1371/journal.pone.0284277 (PMC10096290; doi:10.1371/journal.pone.0284277)
Supplement: S6 Table — (DOCX) [file pone.0284277.s009.docx]

**S6 Table: Amplification status of 1311*fea*2.1 in 197 germplasm**

| S.No. | Genotype | Kernel Row Number | Amplification status of 1311fea2.1 primer |
| --- | --- | --- | --- |
| 1 | PML-2 | 18 | + |
| 2 | PML-3 | 18 | + |
| 3 | PML-4 | 20 | + |
| 4 | PML-5 | 12 | - |
| 5 | PML-6 | 18 | + |
| 6 | PML-7 | 12 | - |
| 7 | PML-8 | 18 | + |
| 8 | PML-9 | 20 | + |
| 9 | PML-11 | 18 | + |
| 10 | PML-12 | 18 | + |
| 11 | PML-13 | 20 | + |
| 12 | PML-14 | 20 | + |
| 13 | PML-15 | 14 | - |
| 14 | PML-17 | 18 | + |
| 15 | PML-18 | 14 | - |
| 16 | PML-19 | 20 | + |
| 17 | PML-20 | 14 | - |
| 18 | PML-21 | 14 | - |
| 19 | PML-23 | 14 | + |
| 20 | PML-24 | 14 | - |
| 21 | PML-25 | 14 | - |
| 22 | PML-26 | 14 | - |
| 23 | PML-27 | 18 | + |
| 24 | PML-28 | 18 | + |
| 25 | PML-29 | 16 | - |
| 26 | PML-30 | 14 | - |
| 27 | PML-31 | 14 | - |
| 28 | PML-32 | 14 | - |
| 29 | PML-33 | 18 | + |
| 30 | PML-34 | 14 | - |
| 31 | PML-36 | 18 | + |
| 32 | PML-37 | 18 | + |
| 33 | PML-40 | 16 | - |
| 34 | PML-43 | 14 | - |
| 35 | PML-44 | 14 | - |
| 36 | PML-45 | 16 | - |
| 37 | PML-46 | 12 | - |
| 38 | PML-47 | 12 | - |
| 39 | PML-49 | 18 | + |
| 40 | PML-50 | 20 | + |
| 41 | PML-51 | 12 | - |
| 42 | PML-53 | 14 | - |
| 43 | PML-54 | 22 | + |
| 44 | PML-54-3 | 14 | - |
| 45 | PML-56 | 16 | - |
| 46 | PML-58 | 18 | + |
| 47 | PML-60 | 22 | + |
| 48 | PML-62 | 14 | - |
| 49 | PML-63 | 20 | + |
| 50 | PML-64 | 14 | - |
| 51 | PML-65 | 18 | + |
| 52 | PML-66 | 18 | + |
| 53 | PML-68 | 20 | + |
| 54 | PML-72 | 12 | - |
| 55 | PML-73 | 18 | + |
| 56 | PML-76 | 12 | - |
| 57 | PML-77 | 16 | + |
| 58 | PML-78 | 18 | + |
| 59 | PML-80 | 14 | - |
| 60 | PML-81 | 18 | + |
| 61 | PML-82 | 20 | + |
| 62 | PML-84 | 12 | - |
| 63 | PML-85 | 18 | + |
| 64 | PML-87 | 18 | + |
| 65 | PML-88 | 18 | + |
| 66 | PML-90 | 20 | + |
| 67 | PML-92 | 20 | + |
| 68 | PML-93 | 14 | - |
| 69 | PML-94 | 18 | + |
| 70 | PML-95 | 20 | + |
| 71 | PML-96 | 14 | - |
| 72 | PML-97 | 16 | - |
| 73 | PML-98 | 14 | - |
| 74 | PML-100 | 18 | + |
| 75 | PML-102 | 20 | + |
| 76 | PML-105 | 14 | - |
| 77 | PML-111 | 14 | - |
| 78 | PML-115 | 12 | - |
| 79 | PML-116 | 20 | + |
| 80 | D-2295-1 | 14 | - |
| 81 | CDM-582 | 18 | + |
| 82 | C-22 | 20 | + |
| 83 | C-137 | 20 | + |
| 84 | BM-1382 | 18 | + |
| 85 | BM-1406 | 22 | + |
| 86 | PDM-4441 | 14 | - |
| 87 | PDM-4321 | 12 | - |
| 88 | C-2760 | 14 | - |
| 89 | CDM-105 | 18 | + |
| 90 | PDM-59 | 18 | + |
| 91 | CDM-445 | 12 | - |
| 92 | C-99 | 12 | - |
| 93 | CDM-435 | 18 | + |
| 94 | C-2745-1 | 20 | + |
| 95 | C-85 | 20 | + |
| 96 | BM-1440 | 18 | + |
| 97 | C-85 | 18 | + |
| 98 | C-142 | 20 | + |
| 99 | CDM-330 | 18 | + |
| 100 | PDM-4241 | 22 | + |
| 101 | C-96 | 14 | - |
| 102 | C-71 | 16 | + |
| 103 | C-2874-4 | 12 | - |
| 104 | CDM-201 | 14 | - |
| 105 | C-70 | 18 | + |
| 106 | C-96-1 (483) | 20 | + |
| 107 | PDM-4641-1 | 22 | + |
| 108 | C-46 | 20 | + |
| 109 | BLSB-5 | 18 | + |
| 110 | C-2752 | 18 | + |
| 111 | C-147 | 18 | + |
| 112 | HKI-1105-1 | 18 | + |
| 113 | DIM-2048 | 18 | + |
| 114 | DIM-312 | 20 | + |
| 115 | PDM-4341 | 14 | - |
| 116 | C-141 | 12 | - |
| 117 | C-113 | 14 | - |
| 118 | C-82 | 12 | - |
| 119 | C-2765 | 18 | + |
| 120 | DIM-334-A-1 | 12 | - |
| 121 | DDM-313 | 12 | - |
| 122 | DMM-270-L | 18 | + |
| 123 | C-2791 | 18 | + |
| 124 | PDM-115-1 | 14 | - |
| 125 | DIM-310-R-1 | 20 | + |
| 126 | PDM-10 | 22 | + |
| 127 | BGD-48/4 | 20 | + |
| 128 | D-2332-4 | 20 | + |
| 129 | PDM-24-4 | 20 | + |
| 130 | C-139 | 20 | + |
| 131 | C-12 | 18 | + |
| 132 | D-2282-1 | 14 | - |
| 133 | CDM-550 | 16 | + |
| 134 | DD-2313-B-1 | 20 | + |
| 135 | C-10 | 18 | + |
| 136 | BM-1472 | 18 | + |
| 137 | PDM-4251 | 20 | + |
| 138 | CDM-554 | 20 | + |
| 139 | KRN-114 | 18 | + |
| 140 | C-113 | 18 | + |
| 141 | DIM-320-2 | 18 | + |
| 142 | DIM-316 | 20 | + |
| 143 | C-84 | 20 | + |
| 144 | C-18 | 22 | + |
| 145 | C-2809-1-2 | 12 | - |
| 146 | C-83 | 18 | + |
| 147 | CDM-320-1 | 18 | + |
| 148 | D-2386-B-2 | 20 | + |
| 149 | BLSB-7 | 20 | + |
| 150 | D-2569-A | 18 | + |
| 151 | LM-14/16-R | 20 | + |
| 152 | 21100388 | 20 | + |
| 153 | 21100403 | 14 | - |
| 154 | Ra-22 | 20 | + |
| 155 | 21100387 | 18 | + |
| 156 | 21100385 | 18 | + |
| 157 | 21100393 | 12 | - |
| 158 | 21100390 | 18 | + |
| 159 | 21100389 | 20 | + |
| 160 | PDM 6555 | 14 | - |
| 161 | AI-564 | 14 | - |
| 162 | PML-18-3 | 18 | + |
| 163 | PML-68 (W) | 18 | + |
| 164 | 21100404 | 20 | + |
| 165 | AI-459 | 16 | - |
| 166 | PDH-1312 | 16 | + |
| 167 | AI-581 | 18 | + |
| 168 | AI-577 | 12 | - |
| 169 | AI-559 | 18 | + |
| 170 | AI-562 | 20 | + |
| 171 | AI-579 | 14 | - |
| 172 | AI-582 | 22 | + |
| 173 | AI-566 | 20 | + |
| 174 | AI-567 | 20 | + |
| 175 | AI-571 | 18 | + |
| 176 | BM-1498 | 18 | + |
| 177 | AI-572 | 22 | + |
| 178 | AI-570 | 20 | + |
| 179 | AI-563 | 18 | + |
| 180 | AI-573 | 14 | - |
| 181 | CML-596 | 14 | - |
| 182 | AI-573 | 18 | + |
| 183 | AI-569 | 18 | + |
| 184 | AI-561 | 20 | + |
| 185 | AI-1523 | 12 | - |
| 186 | AI-560 | 18 | + |
| 187 | AI-568 | 16 | + |
| 188 | AI-558 | 20 | + |
| 189 | PDM-4591 (W) | 14 | - |
| 190 | AI-576 | 12 | + |
| 191 | AI-574 | 18 | + |
| 192 | AI-556 | 16 | + |
| 193 | AI-565 | 14 | - |
| 194 | BM-1500 | 12 | - |
| 195 | BM-1224 | 18 | + |
| 196 | BM-1567 | 18 | + |
| 197 | BM-1589 | 20 | + |
