# Supplementary material for: Designed and validated novel allele-specific primer to differentiate Kernel Row Number (KRN) in tropical field corn
Source: PLoS One. 2023 Apr 12;18(4):e0284277. doi: 10.1371/journal.pone.0284277 (PMC10096290; doi:10.1371/journal.pone.0284277)
Supplement: Attachment — Submitted filename: Response to reviewer.docx [file pone.0284277.s010.docx]

**Designed and Validated Novel Allele-Specific Primer to Differentiate Kernel Row Number (KRN) in Tropical Field Corn**

**Response to reviewer**

| S.No | Query | Answer |
| --- | --- | --- |
| 1 | Provide pedigree information of 45 inbred lines | Provided in supplementary table 3 |
| 2 | Tables 1 and 2 were mentioned in the results section but could not be found | It is available in the table section |
| 3 | Validation of large number of diverse genotypes is required | We validated 197 genotypes for the association of primer with the trait. correlation and regression analysis was done and presented in table 4 |
| 4 | The authors should discuss how the allelic variation in fea2 gene is controlling the KRN trait in corn. | A few points added in the discussion |
| 5 | There is not much background information regarding previously reported QTLs/genes for KRN in corn | Provided in introduction |
