# Supplementary material for: Designed and validated novel allele-specific primer to differentiate Kernel Row Number (KRN) in tropical field corn
Source: PLoS One. 2023 Apr 12;18(4):e0284277. doi: 10.1371/journal.pone.0284277 (PMC10096290; doi:10.1371/journal.pone.0284277)
Supplement: Attachment — Submitted filename: Response to reviewer - 1.docx [file pone.0284277.s011.docx]

**Designed and Validated Novel Allele-Specific Primer to Differentiate Kernel Row Number (KRN) in Tropical Field Corn**

**Response to reviewer #3**

| S.No | **Query** | **Answer** |
| --- | --- | --- |
| 1 | In this manuscript, the authors simply selected a candidate gene fea2.1 and sequenced the gene from two genotypes with variation in kernel row number. | We have not simply selected the fea2.1 sequence. There was a purpose to understand the allelic variation of this gene in tropical germplasm. After a thorough review, 13 gees along with fea2 were selected for one particular project. Among the sequence variation, fea2.1 showed SNP that may possibly differentiate high and Low KRN in maize |
| 2 | The authors mentioned next generation sequencing method several times in this manuscript (page 12, 14). You did not use the next-generation sequence-based approach as mentioned in page 14. In real cases, they just used Sanger’s sequencing method to detect the difference between two alleles. This should be changed in their revised manuscript. | We used the NGS method with the Illumina platform to sequence the amplicons. We have records for that. Why do we have to report otherwise when we don’t have evidence?  Hence no question of changing the methodology |
| 3 | Presence and absence is not the surest way to validate the association between SNP and trait. In this manuscript, they could identify the 694 bp alleles in AI 536 but no amplification in AI 535. This no amplification could be due to human error or PCR artifact. Therefore, it is better to amplify the AI 535 allele so that both homozygotes and heterozygotes can be amplified. Since this is not difficult to do, I am suggesting the authors to do this experiment for acceptance of this manuscript. You may have to design another set of primers for amplifying the AI 535 allele. | This is the crux of the paper. We specifically designed the primer which can differentiate the SNP present among the high and low KRN genotypes. The Mismatch principle given by Drenkard et al. 2000 was used to design a specific primer. There is no question of zygosity. If it 694 amplicon amplifies in low KRN genotypes, then this primer is not valid. So, it has been validated using F2 and other germplasm. |
| 4 | Why did you use AI 535 (lowest KRN, 12) and AI 536 (highest KRN, 18)? Is this the most contrasting pair? | We started with the sequencing of 45 genotypes for fea2. After all required processing, we found that SNP variation at the 1311 position is polymorphic with possible reasoning in AI 535 and AI 536, Low, and High KRN genotypes respectively.  Taking a clue from this we have selected this pair within the available one. Of course, it is not a most contrasting pair. But the observed changes at SNP may be sufficient to make them contrast. On the other hand, we need to prove which may be the most contrasting pair. (Depends on the trait). According to DUS guidelines in maize, they are contrast. |
| 5 | The name of 197 diverse inbred lines and their phenotypic data should be provided. Similarly, there is no information provided for the F2 population used for validation. | Provided in supplementary |
| 6 | Fig. 2, 4, and 6 should go to Supplementary information. If the restriction site is identical, there is no point in keeping as a Figure in the Main Text. | Placed in Supplementary |
| 7 | Table 4 should be deleted. There is no need of giving a single correlation coefficient as a table. It can be described in the text. | Deleted |
| 8 | Introduction: The current rate…….are insufficient: Here it should be a singular verb ‘is’ | Attended |
| 9 | Page 12: use one decimal for PCV and GCV (tables and Text); Heritability should be revised as 0.99, 0.99, and 0.93 (in table and text). | Revised accordingly |
| 10 | Please put space between two words: example at1311 should be at 1311; 542were should be 542 were. There are also other places which should be checked. 1saghaimaroof55?, 2019-20and etc. | Attended |
| 11 | Page: 14: ‘he proposed’ should be ‘they proposed’ since there are multiple authors. | Changed accordingly |
| 12 | Page 16: ‘mismatch principles’ should be ‘mismatched principle’. | Attended |
| 13 | Page 18-19; subheading ‘Conventional primers to locate the SNPs at 1311 nucleotide position’ and ‘Specific primer to locate the SNPs at 1311 nucleotide position’ should be revised since you did not do it for locating the SNPs. | Replaced the word ‘locate’ with ‘differentiate’ |
| 14 | The authors may be requested to put line number in the revised manuscript. | Numbered |
