# Supplementary material for: Designed and validated novel allele-specific primer to differentiate Kernel Row Number (KRN) in tropical field corn
Source: PLoS One. 2023 Apr 12;18(4):e0284277. doi: 10.1371/journal.pone.0284277 (PMC10096290; doi:10.1371/journal.pone.0284277)
Supplement: Attachment — Submitted filename: Editors comments-2.docx [file pone.0284277.s013.docx]

# Answer to the Editor’s comments

| S.No | Comments | Justification |
| --- | --- | --- |
| 1 | You mentioned in response to Query no. 1 that “After a thorough review, 13 genes along with fea2 were selected for one particular project. Among the sequence variation, fea2.1 showed SNP that may possibly differentiate high and Low KRN in maize”. I don’t see any where (Materials and Methods or Results), you discussed about it. If that is the case, you should show those genes and sequences to support your statemen | In the result we have added as:  *Differentiation of high and low KRN genotype through allele-specific primer*  In the present study, the challenge was to differentiate high and low KRN genotypes based on the single-nucleotide difference (A to T transversion) present at the 1311 nucleotide position of the tested genotype.  In the Material method section, we added:  *Conventional primers to differentiate the SNPs at 1311 nucleotide position*  There was as an A to T transversion at the 1311 nucleotide position of tested genotypes which probably differentiates high KRN genotypes from low KRN. |
| 2 | I agree with the reviewer 3 that amplicon sequencing is not a next generation sequencing (NGS) technology. Next-generation sequencing (NGS) is a massively parallel sequencing technology that offers ultra-high throughput, scalability, and speed.  Some references are: (a) Goodwin et al. 2016. Coming of age: ten years of next generation sequencing technologies. Nature Review Genetics 2016, Volume 17, pp 333-351; (b) Slatko et al. 2018. Overview of Next Generation Sequencing Technologies. Current Protocols in Mol Biol 2018 Vol 122(1):e59. | We agree with the reviewer and correction has been made by replacing the term next-generation sequencing with amplicon sequencing which was performed on the Illumina platform. |
| 3 | Line #105: You targeted coding sequences of the gene. Could you please discuss about the gene. For ex. Length, no. of exons, and other molecular characteristics. Is 1842 bp amplicon from a single exon? | Modified as: The exploration of the sequencing technology enables the identification of variations linked to the trait at the single-nucleotide level. Since the inbred lines used in the present investigation are of tropical origin, it is hypothesized that the KRN gene, *fea2,* does have allelic variation. The *fea2* is 2489 bp in length and has one intron and two exons that code for 593 amino acids. The *fea2* is also anticipated to have extra-cytoplasmic LRRs, a transmembrane domain, and a short cytoplasmic tail, which acts as a receptor at the plasma membrane (Fumio Taguchi-Shiobara et al. 2001). To understands the allelic variation, the coding sequence (CDS) of *fea*2 was targeted to capture the SNPs among AI 535 and AI 536. The PCR amplicon of the 1842 bp (Fig. 2) obtained from the CDS region that includes 5′ UTR region (56 bp) was sequenced in both AI 535 and AI 536 lines. |
| 4 | Supplementary files and Tables were not cited in the Text. It was hard to track these information because these tables and figures were not placed in sequence. I will suggest to put all supplementary table in one file and all suppl figures in another file with suppl table or suppl. Figure 1 first and so on. | The suggestions have been incorporated and made all the supplementary tables and figures in chronological order as cited in the text. |
| 5 | In supplementary Table 4, please expand the abbreviations in the footnotes. IPCA1, IPCA2, ASVi, ASVi rank etc. | Abbreviations were added to the table which has been renamed as supplementary table 1 |
| 6 | Query no. 5: Please mention which suppl table contains information about 197 diverse inbred lines. | The diverse inbred line of 197 has already been mentioned in the supplementary table 6 as you suggested. |
| 7 | Since the reviewers will first look at your responses before the manuscript, it is advisable to give a detailed response regarding the changes made in the manuscript. | We have done our best to answer all the queries. The incorporation of the details and their explanation has been done in the track change mode and the same is being submitted for your kind reference and perusal. |
|  | New Comments |  |
| 8 | Line 18 the reference to next…… revised | Revised |
| 9 | Line 21-22 | Revised the methodology and findings |
| 10 | Line 27-28 Delete | Deleted “The utilization…….genetic gain |
| 11 | Line 29 | Deleted “easy” |
| 12 | Line 32-33 | Deleted “ as the KRN … fairly simple |
| 13 | Line 55-56 | Deleted “ studying………..markers |
| 14 | Line 99 | Modified |
| 15 | Line 105 | Modified and removed ‘s’ from understands |
| 16 | Line 374 | Table 4 removed |
| 17 | Advised to give details to supplementary table with caption | Given all 6 tables captions |
| 18 | Please ensure that you refere to 2,4,6, fig | It was renamed and corrected |
| 19 | Please include Table 4 | Included plz |
| 20 | Upload without file with track change | uploaded |
